# Risk factors for blood transfusion in traumatic and postpartum hemorrhage patients: Analysis of the CRASH-2 and WOMAN trials

**DOI:** 10.1371/journal.pone.0233274

**Published:** 2020-06-03

**Authors:** David A. Kolin, Haleema Shakur-Still, Adenike Bello, Rizwana Chaudhri, Imelda Bates, Ian Roberts

**Affiliations:** 1 Department of Population Health, Clinical Trials Unit, London School of Hygiene & Tropical Medicine, London, England, United Kingdom; 2 Department of Obstetrics and Gynecology, University of Ibadan, Ibadan, Nigeria; 3 Department Obstetrics and Gynecology, Rawalpindi Medical University, Rawalpindi, Pakistan; 4 Liverpool School of Tropical Medicine, Liverpool, England, United Kingdom; Institute of Experimental Hematology and Transfusion Medicine, University Clinic of Bonn, GERMANY

## Abstract

**Background:**

Hemorrhage is a leading cause of death after trauma and childbirth. In response to severe hemorrhage, bleeding patients often receive transfusions of red blood cells, plasma, platelets, or other blood components. We examined risk factors for transfusion in acute severe bleeding in two trials of over 20,000 patients to better understand factors associated with transfusion likelihood.

**Study design and methods:**

We conducted a cohort analysis of data from the CRASH-2 and WOMAN trials, two multinational trials that recruited patients with traumatic and postpartum hemorrhage, respectively. For each trial, we examined the effect of 10 factors on blood transfusion likelihood. Univariate and multivariate Poisson regressions were used to analyze the relationship between risk factors and blood transfusion.

**Results:**

Of the 20,207 traumatic hemorrhage patients, 10,232 (51%) received blood components. Of the 20,060 women with postpartum hemorrhage, 10,958 (55%) received blood components. For patients who suffered from traumatic hemorrhage, those greater than three hours from injury to hospitalization were more likely to be transfused (ARR 1.37; 95% CI, 1.20–1.56). Postpartum hemorrhage patients had an increased likelihood of transfusion if they gave birth outside the hospital (ARR 1.30; 95% CI 1.22–1.39), gave birth more than three hours before hospitalization (ARR 1.09; 95% CI 1.01–1.17), had a Caesarean section (ARR 1.16; 95% CI 1.08–1.25), and if they had any identifiable causes of hemorrhage other than uterine atony.

**Conclusion:**

Several risk factors are associated with an increased likelihood of transfusion in traumatic and postpartum hemorrhage patients. Altering modifiable factors, by reducing time from injury or childbirth to hospitalization, for example, might be able to reduce transfusions and their complications.

**Trial registration:**

CRASH-2 is registered as ISRCTN86750102, ClinicalTrials.gov NCT00375258 and South African Clinical Trial Register DOH-27–0607–1919. WOMAN is registered as ISRCTN76912190, ClinicalTrials.gov NCT00872469, PACTR201007000192283, and EudraCT number 2008-008441-38.

## Introduction

World-wide about 10% of deaths are due to trauma. Deaths from trauma disproportionately affect people in lower income countries, with more than 90% of deaths due to injury occurring in low- and middle-income countries [1,2]. Approximately 40% of trauma deaths are due to bleeding, making hemorrhage the leading cause of death following injury [3]. Appropriate management of traumatic bleeding includes rapid identification of bleeding sources, followed by immediate measures to stem blood loss and restore hemodynamic stability [4]. Trauma patients who require massive transfusion or develop coagulopathy have poor outcomes [5,6].

Postpartum hemorrhage, typically defined as blood loss ≥500 mL within 24 hours of birth, is also a substantial cause of mortality. Postpartum hemorrhage occurs in about 6% of all childbirths and is responsible for up to 100,000 deaths each year (approximately one quarter of all maternal deaths) [7,8]. Risk factors for postpartum hemorrhage include prolonged third stage of labor, multiple births, fetal macrosomia, episiotomy and previous postpartum hemorrhage [9,10]. Almost all (99%) maternal hemorrhage deaths are in low and middle income countries [11].

Blood transfusions are often given to replace blood lost after traumatic and postpartum hemorrhage [12,13]. However, in low-income countries there is a limited supply of safe blood for transfusion [14]. Due to transfusion transmitted infections, low donation rates, sub-optimal management of blood stocks and inadequate distribution of blood, many low- and middle-income countries are unable to meet transfusion demand [15,16]. To better understand how both hemodynamic and non-hemodynamic risk factors influence transfusion likelihood, we assessed the risk factors associated with likelihood of transfusion in two large, previously-published multinational trials.

## Materials and methods

### Study design

The institutional review board of the London School of Hygiene & Tropical Medicine approved this study. This is a retrospective analysis of two randomized controlled trials. The details of the trial protocol and approval are published and publicly available. Data were analyzed anonymously.

The CRASH-2 trial was a randomized placebo-controlled trial of tranexamic acid in 20,207 bleeding trauma patients conducted in 274 hospitals in 40 countries [17]. Adult trauma patients with or at risk of acute significant hemorrhage, defined as hemorrhage within 8 hours of the injury, were randomly allocated to receive tranexamic acid or placebo. Baseline data were recorded prior to randomization. Outcome data were recorded at discharge from the randomizing hospital, 28 days after injury, or death, whichever came first [18].

The WOMAN trial was a randomized double-blind, placebo-controlled trial of tranexamic acid in 20,060 women with postpartum hemorrhage that was conducted in 193 hospitals in 21 countries. Women aged 16 years or older with a diagnosis of postpartum hemorrhage after vaginal birth or caesarean section were randomly allocated to tranexamic acid or placebo. The diagnosis of postpartum hemorrhage was clinical (estimated blood loss ≥500 mL after vaginal birth or ≥1000 mL after caesarean section). Baseline data were recorded prior to randomization. Outcome data were recorded at discharge from the randomizing hospital, 42 days after giving birth or death, whichever came first [19,20]. Neither the CRASH-2 trial nor the WOMAN trial found a significant difference in the incidence of transfusion by trial arm.

For the cohort analyses, the study outcome was defined as any blood component transfusion (whole or packed red blood cells, fresh frozen plasma, and other blood components). The exposure country income level, measured by gross national income per capita for each country, was classified according to the World Bank 2017 system, using the Atlas method (see appendix). The World Bank’s method for classifying country income levels allows for inter-country income comparisons. The Atlas method reduces fluctuations in exchange rates by using a three-year moving average, price adjusted conversion factor [21]. For trauma patients, potential risk factors were systolic blood pressure, age, sex, Glasgow Coma Score, injury type, respiratory rate, heart rate, capillary refill time, use of tranexamic acid, and time from injury to randomization (a proxy for the time elapsed from injury to the diagnosis of hemorrhage) [22,23]. For women with postpartum hemorrhage, potential risk factors were systolic blood pressure, age, hospital childbirth, type of birth, placenta fully expelled, primary cause of hemorrhage, estimated blood loss, clinical signs of hemodynamic instability, use of tranexamic acid, and time from childbirth to randomization (a proxy for the time elapsed from delivery to the diagnosis of postpartum hemorrhage) [24,25]. The adjustments for use of tranexamic acid were used as a sensitivity analysis to determine if the randomized intervention also acted as a risk factor.

### Statistics

For statistical analyses, we used Stata 14 and R, version 3.4.3. *A priori*, we created subgroups for risk factors and outcomes based on commonly-used strata for each factor. To show the frequency distribution of blood component transfusions, we prepared histograms of blood transfusion volumes by country income group. We cross tabulated exposures and outcome, including both missing and present data. For variables with more than 1% missing data, we explored baseline characteristics of participants with missing data to understand how participants with and without missing data differed. We conducted univariate analyses to examine risk factors for transfusion. To avoid the possibility of failed convergence with the log-binomial model, we used a robust, correlation-adjusted Poisson model to estimate relative risks [26,27]. Univariate robust effects Poisson models were used to calculate crude risk ratios, adjusted for clustering of subjects by hospital. A backwards approach was used to create a multivariate correlation-adjusted Poisson model. Variables were individually removed from the model to assess for confounding and collinearity. To produce our multivariate model, we controlled for all variables of interest, including factors indicative of bleeding severity. Hemodynamic factors were determined based on a pathophysiological understanding of the mechanisms of hemorrhage. For the CRASH-2 trial, variables reflective of bleeding severity were systolic blood pressure, Glasgow Coma Score, respiratory rate, heart rate, and capillary refill time. For the WOMAN trial, variables reflective of hemodynamic status were systolic blood pressure, estimated blood loss, and clinical signs of hemodynamic instability. We also examined the association between country income level and the receipt of single unit blood component transfusions to better understand how factors such as low blood supplies in low income countries affect the receipt of blood transfusions.

## Results

### Trauma patients

**Fig 1:**
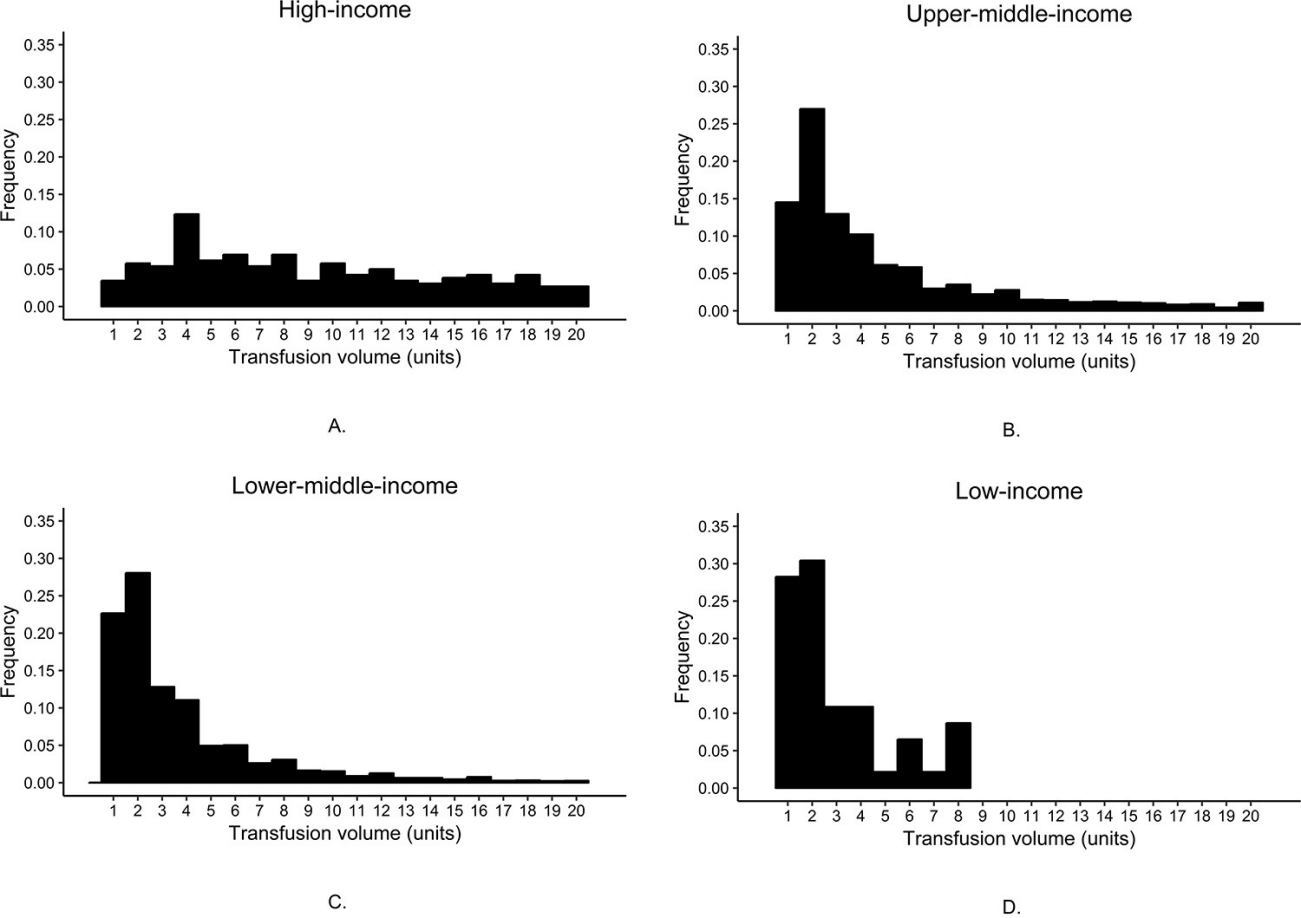
The frequency of blood transfusion volumes differs by country income group in traumatic hemorrhage patients: high-income (A), upper-middle-income (B), lower-middle-income (C), and low-income (D).

Table 1 shows the characteristics of the 20,207 participants included in the CRASH-2 trial. 10,232 (51%) participants were transfused with blood components. Average time from injury to randomization was 3.3 hours, with 37% (7,478) in the ≤1 hour group. In this cohort, 16,935 (84%) participants were male, 13,692 (68%) had systolic blood pressure ≥90 mmHg, and 12,132 (60%) were recruited from lower-middle-income countries. The average age of study participants was 34.6 years. The frequencies of blood transfusion for high-income, upper-middle-income, lower-middle-income, and low-income countries were 80%, 54%, 48%, and 72%, respectively. For those who were transfused with blood components, the volume of blood components transfused differed by country income group (Fig 1). With decreasing country income level, the frequency of single unit blood transfusions increased: high-income (3%), upper-middle-income (13%), lower-middle-income (22%), and low-income (28%). Patients with blunt and penetrating injury were more likely to receive a transfusion than patients with blunt injury alone (RR 1.18; 95% CI 1.03–1.36). In univariate analyses, age and sex did not affect transfusion likelihood. Compared with patients with systolic blood pressure ≥90 mmHg, those with a systolic blood pressure ≤90 mmHg had an increased risk of blood transfusion. Of the risk factors included in this study, only one had >1% missing data. 611 (3%) patients were missing data on capillary refill time, and they had a higher risk of transfusion than participants without missing data (RR 1.12; 95% CI 1.04–1.20). Only 80 participants (<1%) had missing data on blood component transfusion.

**Table 1:** Analysis of risk factors for blood transfusion in traumatic hemorrhage patients.

Variable	Total	Blood component transfusion (% transfused)	Crude risk ratio (95% CI)	Adjusted risk ratio* (95% CI)
Country income group^†^				
High	417 (2.1%)	381 (80.0%)	1	1
Upper-middle	7594 (37.6%)	4039 (53.5%)	0.65 (0.58–0.72)	0.78 (0.66–0.91)
Lower-middle	12132 (60.0%)	5766 (47.9%)	0.58 (0.49–0.68)	0.66 (0.55–0.80)
Low	64 (0.3%)	46 (71.9%)	0.87 (0.82–0.91)	0.73 (0.63–0.85)
Age (years)				
<25	5638 (27.9%)	2810 (50.0%)	1	1
25–34	6093 (30.2%)	3105 (51.1%)	1.02 (0.97–1.07)	1.01 (0.97–1.05)
35–44	3816 (18.9%)	1953 (51.4%)	1.03 (0.97–1.09)	1.00 (0.96–1.04)
>44	4656 (23.0%)	2363 (51.0%)	1.02 (0.94–1.10)	0.98 (0.92–1.04)
Unknown	4 (<0.1%)			
Sex				
Male	16935 (83.8%)	8493 (50.4%)	1	1
Female	3271 (16.2%)	1738 (53.4%)	1.06 (0.99–1.14)	1.05 (1.00–1.10)
Unknown	1 (<0.1%)			
Time since injury (h)				
≤1	7478 (37.0%)	3248 (43.6%)	1	1
1–3	6051 (30.0%)	3263 (54.1%)	1.24 (1.08–1.43)	1.25 (1.12–1.40)
>3	6667 (33.0%)	3715 (56.0%)	1.28 (1.10–1.50)	1.37 (1.20–1.56)
Unknown	11 (<0.1%)			
Injury type				
Blunt	11189 (55.4%)	5557 (49.8%)	1	1
Penetrating	6552 (32.4%)	3229 (49.5%)	0.99 (0.88–1.12)	1.01 (0.92–1.11)
Blunt and penetrating	2466 (12.2%)	1446 (59.0%)	1.18 (1.03–1.36)	1.12 (1.00–1.25)
Systolic blood pressure (mmHg)				
≥90	13692 (67.8%)	5742 (42.1%)	1	1
76–89	3312 (16.4%)	2159 (65.5%)	1.55 (1.41–1.72)	1.41 (1.29–1.53)
≤75	3174 (15.7%)	2110 (73.5%)	1.75 (1.57–1.95)	1.53 (1.41–1.65)
Unknown	29 (0.1%)			
Glasgow Coma Score (total)				
Mild (13–15)	13842 (68.5%)	6499 (47.1%)	1	1
Moderate (9–12)	2704 (13.4%)	1536 (57.0%)	1.21 (1.04–1.41)	1.05 (0.92–1.19)
Severe (3–8)	3638 (18.0%)	2190 (60.5%)	1.28 (1.13–1.46)	1.11 (1.00–1.24)
Unknown	23 (0.1%)			
Respiratory rate (per min)				
<10	305 (1.5%)	207 (68.1%)	1.40 (1.11–1.77)	1.02 (0.76–1.38)
10–29	16791 (83.1%)	8117 (48.5%)	1	1
>29	2920 (14.5%)	1788 (61.6%)	1.27 (1.16–1.39)	1.08 (1.00–1.17)
Unknown	191 (1.0%)			
Heart rate (beats per min)				
<77	1746 (8.6%)	742 (42.8%)	1.15 (1.05–1.26)	1.06 (0.98–1.15)
77–91	3497 (17.3%)	1298 (37.2%)	1	1
92–107	5102 (25.3%)	2279 (44.9%)	1.21 (1.12–1.30)	1.16 (1.09–1.24)
>107	9725 (48.1%)	5804 (59.9%)	1.61 (1.45–1.79)	1.37 (1.25–1.51)
Unknown	137 (0.7%)			
Central capillary refill time (s)				
≤2	6838 (33.8%)	3017 (44.3%)	1	1
3–4	9387 (46.5%)	4746 (50.7%)	1.14 (0.95–1.38)	1.05 (0.90–1.22)
>4	3371 (16.7%)	2124 (63.3%)	1.43 (1.20–1.70)	1.13 (0.96–1.32)
Unknown	611 (3.0%)			

*Adjusted variables include age, sex, time since injury, injury type, systolic blood pressure, Glasgow Coma Score, respiratory rate, heart rate, central capillary refill time, and use of tranexamic acid

^†^Income group was defined according to the World Bank’s June 2017 classification system

To control for confounding, we calculated adjusted risk ratios (ARR). Relative to patients in high-income countries, patients in lower-middle-income countries were less likely to receive a blood component transfusion (ARR 0.66; 95% CI 0.55–0.80). The decreased frequency of blood transfusion in lower income countries was evident even for blood transfusions greater than one unit (Table 2). Analyses of single unit blood transfusion indicated increasing likelihood of single unit blood transfusion with decreasing country income level. Relative to patients in high-income countries, traumatic hemorrhage patients in low-income countries had over 10 times the risk of single unit blood transfusion (ARR 10.04; 95% CI 4.06–24.82).

**Table 2:** Adjusted risk ratios for blood transfusion by country income group after traumatic hemorrhage.

Country income group^†^	Crude risk ratio (95% CI)	Adjusted risk ratio (95% CI)*
Outcome: >1 unit transfusion		
High	1	1
Upper-middle	0.57 (0.48–0.68)	0.70 (0.57–0.86)
Lower-middle	0.46 (0.39–0.55)	0.55 (0.45–0.67)
Low	0.64 (0.60–0.68)	0.54 (0.45–0.64)
Outcome: 1 unit transfusion		
High	1	1
Upper-middle	3.28 (1.04–10.31)	4.87 (1.85–12.80)
Lower-middle	4.91 (1.72–14.03)	6.24 (2.50–15.59)
Low	9.34 (3.35–26.03)	10.04 (4.06–24.82)

*Adjusted variables include age, sex, time since injury, injury type, systolic blood pressure, Glasgow Coma Score, respiratory rate, heart rate, central capillary refill time, and use of tranexamic acid

^†^Income group was defined according to the World Bank’s June 2017 classification system

After adjustment, there was some evidence that female sex was associated with an increased risk of blood transfusion (ARR 1.05; 95% CI 1.00–1.10). The adjusted relative risks for time from injury to randomization were 1.25 (95% CI, 1.12–1.40) in patients in the 1–3 hour group and 1.37 (95% CI, 1.20–1.56) for patients in the >3 hour group. Adjusting for all variables, blunt and penetrating injury were associated with a 12% greater risk of transfusion when compared to blunt injury alone. Amongst hemodynamic factors, the greatest likelihood of transfusion was for patients with systolic blood pressure ≤75 (ARR 1.53; 95% CI 1.41–1.65) and for patients with heart rate >107 (ARR 1.37; 95% CI 1.25–1.51).

### Women with postpartum hemorrhage

**Fig 2:**
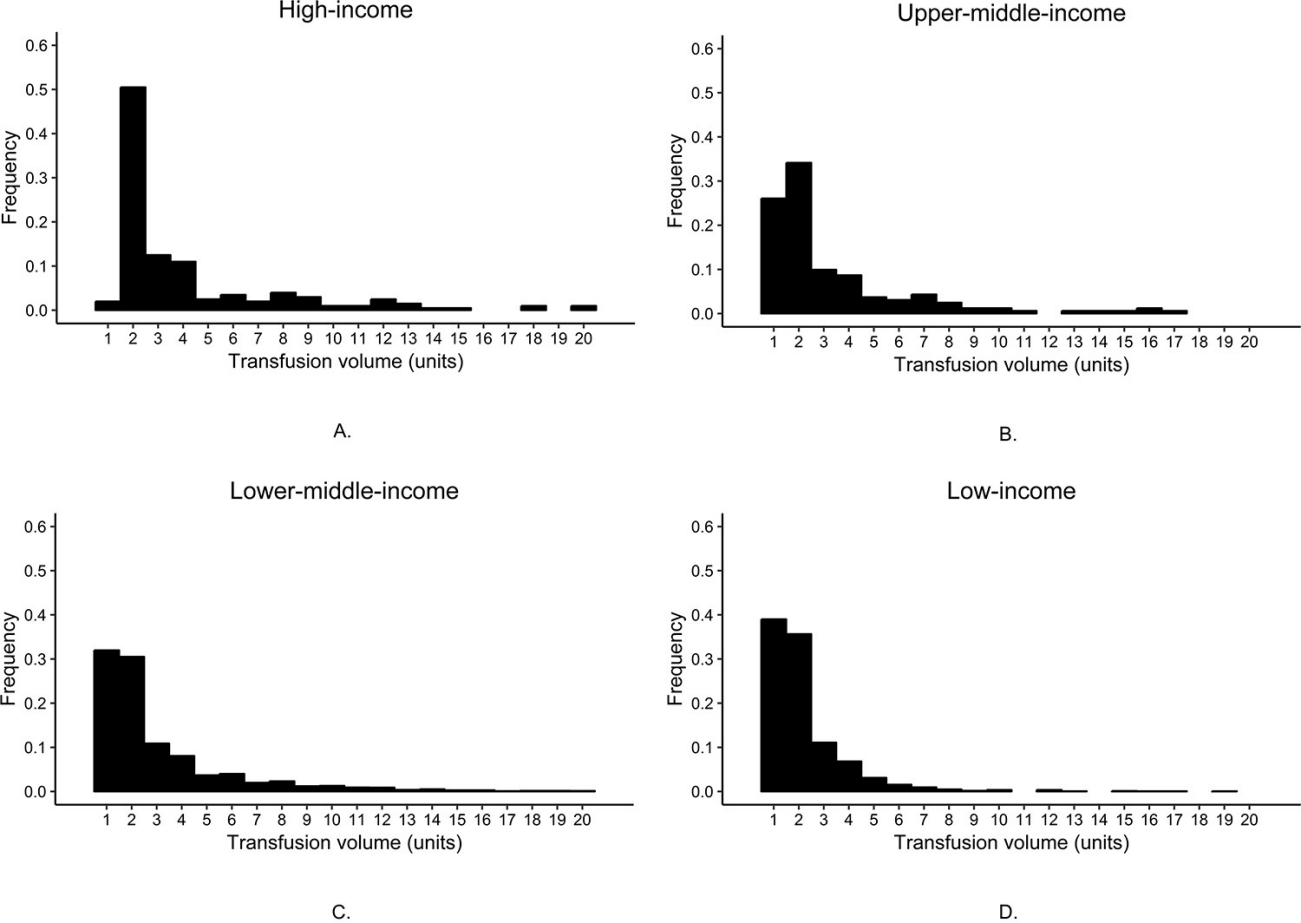
The frequency of blood transfusion volumes differs by country income group in postpartum hemorrhage patients: high-income (A), upper-middle-income (B), lower-middle-income (C), and low-income (D).

Table 3 shows data for the 20,060 women enrolled in the WOMAN trial. Of the study participants, 10,958 (55%) received blood component transfusions. Time from childbirth to randomization was 3.4 hours, on average, with 48% of patients (9,585) in the ≤1 hour group. 88% (17,625) of women delivered in the hospital. The frequencies of blood transfusion for patients in high-income, upper-middle-income, lower-middle-income, and low-income countries were 36%, 30%, 61%, and 40%, respectively. For women transfused with blood components, transfusion volume differed by country income group (Fig 2). With decreasing country income level, the frequency of single unit blood component transfusions increased: high-income (2%), upper-middle-income (25%), lower-middle-income (32%), and low-income (39%). Age was a risk factor for blood transfusion in women with postpartum hemorrhage. Relative to patients in the ≤25 year age category, patients in the >33 year age category had a 24% increased risk of blood transfusion. Childbirth outside of the hospital increased the risk of blood component transfusion (RR 1.54; 95% CI 1.41–1.67). If time from delivery to randomization was >3 hours, the risk of blood transfusion increased, both for women who delivered outside of the hospital (RR 1.26; 95% CI 1.05–1.52) and women who delivered inside of the hospital (RR 1.14; 95% CI 1.03–1.26). Of the exposures investigated in the WOMAN trial, no risk factor had >1% missing data. In the WOMAN trial, only 43 (<1%) of trial participants had missing outcome data.

**Table 3:** Analysis of risk factors for blood transfusion in postpartum hemorrhage patients.

Variable	Total	Blood component transfusion (% transfused)	Crude risk ratio (95% CI)	Adjusted risk ratio* (95% CI)
Country income group^†^				
High	569 (2.8%)	202 (35.8%)	1	1
Upper-middle	566 (2.8%)	168 (29.7%)	0.83 (0.40–1.72)	0.98 (0.47–2.05)
Lower-middle	14718 (73.4%)	8917 (60.7%)	1.69 (1.36–2.11)	1.55 (1.30–1.85)
Low	4207 (21.0%)	1671 (39.8%)	1.11 (0.86–1.44)	1.06 (0.84–1.33)
Age (years)				
≤25	6856 (34.2%)	3453 (50.5%)	1	1
26–33	9188 (45.8%)	5007 (54.6%)	1.08 (1.03–1.14)	0.98 (0.94–1.02)
>33	4009 (20.0%)	2498 (62.4%)	1.24 (1.16–1.32)	1.01 (0.96–1.06)
Unknown	7 (<0.1%)			
Hospital childbirth				
Yes	17625 (87.9%)	9039 (51.4%)	1	1
No	2432 (12.1%)	1917 (79.0%)	1.54 (1.41–1.67)	1.30 (1.22–1.39)
Unknown	3 (<0.1%)			
Time from childbirth (h)				
≤1	9585 (47.8%)	4754 (49.7%)	1	1
>1–3	5369 (26.8%)	2892 (54.0%)	1.09 (0.99–1.19)	1.01 (0.94–1.09)
>3	5091 (25.4%)	3303 (65.0%)	1.31 (1.19–1.44)	1.09 (1.01–1.17)
Unknown	15 (<0.1%)			
Type of childbirth				
Vaginal childbirth	14219 (70.9%)	6990 (49.3%)	1	1
Caesarean section	5836 (29.1%)	3965 (68.1%)	1.38 (1.29–1.48)	1.16 (1.08–1.25)
Unknown	5 (<0.1%)			
Placenta fully expelled				
Yes	18105 (90.3%)	9603 (53.2%)	1	1
No	1952 (9.7%)	1353 (69.4%)	1.30 (1.21–1.41)	1.00 (0.95–1.06)
Unknown	3 (<0.1%)			
Primary cause of hemorrhage				
Uterine atony	12784 (63.7%)	6282 (49.2%)	1	1
Surgical trauma or tears	3691 (18.4%)	2107 (57.2%)	1.16 (1.07–1.26)	1.08 (1.01–1.15)
Placenta previa or accreta	1878 (9.4%)	1457 (77.8%)	1.58 (1.44–1.73)	1.19 (1.12–1.27)
Other	1457 (7.3%)	987 (67.9%)	1.38 (1.21–1.57)	1.19 (1.10–1.29)
Undetermined	246 (1.2%)	123 (50%)	1.02 (0.83–1.24)	0.95 (0.82–1.11)
Unknown	4 (<0.1%)			
Systolic blood pressure (mmHg)				
≥90	16203 (80.8%)	8176 (50.5%)	1	1
76–89	2179 (10.9%)	1486 (68.5%)	1.36 (1.23–1.49)	1.07 (1.01–1.13)
≤75	1673 (8.3%)	1295 (77.7%)	1.54 (1.38–1.71)	1.10 (1.04–1.16)
Unknown	5 (<0.1%)			
Estimated blood loss (mL)				
≤500	608 (3.0%)	131 (21.6%)	1	1
>500 to ≤1000	9810 (48.9%)	3820 (39.0%	1.81 (1.37–2.37)	1.69 (1.30–2.20)
>1000 to ≤1500	5714 (28.5%)	3680 (64.5%)	2.99 (2.21–4.05)	2.39 (1.80–3.18)
>1500	3926 (19.6%)	3327 (85.1%)	3.94 (2.88–5.39)	2.84 (2.12–3.78)
Unknown	2 (<0.1%)			
Clinical signs of hemodynamic instability				
No	8200 (40.9%)	3075 (37.5%)	1	1
Yes	11859 (59.1%)	7883 (66.7%)	1.78 (1.50–2.11)	1.43 (1.24–1.64)
Unknown	1 (<0.1%)			

*Adjusted variables include country income group, age, hospital childbirth, time from childbirth, type of childbirth, placenta fully expelled, primary cause of hemorrhage, systolic blood pressure, estimated blood loss, clinical signs of hemodynamic instability, and use of tranexamic acid

^†^Income group was defined according to the World Bank’s June 2017 classification system

After adjustment, there was strong evidence of an increase in transfusion likelihood in lower-middle-income countries (ARR 1.55; 95% CI 1.30–1.85). However, this association disappeared upon analysis of blood transfusions greater than one unit, and all risk ratios decreased in magnitude (Table 4). When single unit blood transfusions were excluded, the adjusted relative risk for lower-middle-income countries was 1.07 (95% CI 0.89–1.28), and there was only evidence of a decrease in risk in low-income countries relative to high-income countries (ARR 0.67; 95% CI 0.49–0.91). Lower country income level was associated with an increased risk of single unit blood transfusion. Patients in lower-middle-income countries had the greatest risk of single unit blood transfusion (ARR 24.40; 95% CI 13.82–43.07).

**Table 4:** Adjusted risk ratios for blood transfusion by country income group after postpartum hemorrhage.

Country income group^†^	Crude risk ratio (95% CI)	Adjusted risk ratio* (95% CI)
Outcome: >1 unit transfusion		
High	1	1
Upper-middle	0.63 (0.34–1.18)	0.84 (0.45–1.57)
Lower-middle	1.18 (0.92–1.51)	1.07 (0.89–1.28)
Low	0.69 (0.49–0.97)	0.67 (0.49–0.91)
Outcome: 1 unit transfusion		
High	1	1
Upper-middle	10.46 (3.13–35.00)	9.50 (2.81–32.11)
Lower-middle	27.09 (15.18–48.36)	24.40 (13.82–43.07)
Low	21.89 (11.98–39.99)	19.36 (10.77–34.80)

*Adjusted variables include age, hospital childbirth, time from childbirth, type of childbirth, placenta fully expelled, primary cause of hemorrhage, systolic blood pressure, estimated blood loss, clinical signs of hemodynamic instability, and use of tranexamic acid

^†^Income group was defined according to the World Bank’s June 2017 classification system

After controlling for severity of bleeding there was no association between maternal age and the risk of transfusion. Childbirth outside of the hospital was associated with an increased risk of transfusion (ARR 1.30; 95% CI 1.22–1.39). Adjusted risk ratios for increasing time since childbirth indicated a moderate increase in transfusion likelihood in women >3 hours after childbirth (ARR 1.09; 95% CI 1.01–1.17). While caesarean section increased the risk of transfusion (ARR 1.16; 95% CI 1.08–1.25), incomplete expulsion of the placenta did not (ARR 1.00; 95% CI 0.95–1.06). All identifiable causes of hemorrhage–including surgical trauma or tears, placenta previa or accreta, and other causes of hemorrhage–were associated with an increased likelihood of transfusion relative to uterine atony. Amongst hemodynamic factors, blood loss >1500 mL was associated with the greatest likelihood of transfusion (ARR 2.84; 95% CI 2.12–3.78).

## Discussion

For patients who suffered from traumatic hemorrhage, the only risk factor for transfusion, other than variables indicative of hemodynamic status, is time since injury. Patients with postpartum hemorrhage have an increased likelihood of transfusion with childbirth outside of the hospital, increased time since childbirth, Caesarean section, and all identifiable causes of hemorrhage other than uterine atony. Our study shows that lower country income level is associated with lower transfusion likelihood in traumatic hemorrhage patients but is associated with higher transfusion likelihood in postpartum hemorrhage patients. In women with postpartum hemorrhage, these results are largely driven by the administration of single unit blood transfusions. In postpartum hemorrhage patients, exclusion of single unit blood transfusions reduces the risk of blood transfusion in all country income levels, relative to high-income countries.

Both studies were large, randomized placebo-controlled trials with minimal missing data. The likelihood of information bias was low for blood transfusion because it is a well-recorded outcome. However, measurement error may have occurred for some variables of interest like systolic blood pressure, raising the possibility of residual confounding.

Both the CRASH-2 and WOMAN trials were designed to assess the effect of tranexamic acid on patient outcomes, rather than to determine the risk factors for transfusion. Both studies did not collect data on some factors of interest, including blood availability and transfusion practices. Country income level was a proxy for these unrecorded factors [28,29]. In future studies, recording data on more specific factors, that vary by country income level, would help reveal why transfusion likelihood differs by country income group.

We used log-link robust Poisson regression in this study for several reasons. First, such models allow for the direct assessment of risk ratios. Odds ratios may have overestimated the effect measures in this study due to the high incidence of blood transfusions. Second, binomial models for estimating relative risks often do not converge [26]. While we opted for a robust Poisson regression model in this study, the use of Poisson regression is known to produce conservative confidence intervals, suggesting that the true confidence intervals for the risk ratios would likely be narrower than those estimated.[30]

A stepwise approach was taken to generate multivariate Poisson regression models that accounted for confounders. We also adjusted for clustering at the hospital level. Even still, unmeasured confounding may account for some of this study’s results. While we controlled for a number of factors relevant to severity of bleeding, residual confounding may still exist. Importantly, data on hemoglobin level were not obtained for either trial. Hemoglobin level is frequently used as a threshold marker for the initiation of blood transfusions [29,31]. While hemoglobin levels are typically known for patients in high income countries, these data are not always available in low income countries. However, in cases of acute hemorrhage, hemoglobin level is often not an appropriate indicator for transfusion [32].

Instead, hemodynamic factors tend to guide transfusion practices in both trauma and postpartum hemorrhage patients. Studies of blood transfusions in trauma settings suggest that patients with elevated blood pressures and normal heart rates are less likely to receive blood transfusions, including massive transfusions (greater than or equal to ten units of blood) [33,34]. This finding is influenced by the fact that, in settings of acute trauma, decisions to transfuse are often driven by the use of scoring systems like the shock index (defined as heart rate divided by systolic blood pressure). For women with postpartum hemorrhage, the decision to transfuse typically depends on estimated blood loss ≥500 mL, rising pulse, and decreasing blood pressure, as long as a reversible cause of hemorrhage is not confirmed [35]. If a cause of hemorrhage is identified, reversal of the underlying cause of hemorrhage is attempted prior to transfusion. Because hemodynamic variables are used as indicators to initiate blood transfusions in both trauma and postpartum hemorrhage patients, it is not surprising that our findings–that hemodynamically unstable patients are more likely to be transfused– are consistent with previous work.

Likewise, our finding of less frequent large-volume blood transfusions in countries of lower income are consistent with other reports [36–38]. The availability and safety of blood components affects blood transfusion rates. The World Health Organization recommends a blood donation rate of at least 10 units per 1000 people per year. Currently, however, many countries do not meet minimum recommended donation rates. In high-income, upper-middle-income, lower-middle-income, and low-income countries, blood donation rates are 32.1, 14.9, 7.8, and 4.6 donations per 1000 people per year, respectively [15]. Due to low blood donation rates and high population prevalence rates of transfusion-transmissible infections, many low- and middle-income countries are unable to meet transfusion demand.

Differences in anemia may help explain the different transfusion practices in traumatic and postpartum hemorrhage. Anemia is more prevalent in pregnant women than non-pregnant women and is more prevalent in low-income settings than high-income settings [39]. Globally, 33% of non-pregnant women are anemic, and 40% of pregnant women are anemic, with significant variability by region [40,41]. One study, conducted in Southeast Asia, found that 80% of pregnant women and 60% of non-pregnant women were anemic [42]. For postpartum hemorrhage patients in low-income settings, low-volume blood transfusions may be used to simultaneously treat both acute postpartum hemorrhage and prevalent anemia [43–46].

For lower-middle-income countries in the WOMAN trial, limited blood supplies likely led to the frequent administration of single unit blood transfusions, despite the need for higher volume transfusions [47]. Most patients in the lower-middle-income country category were from Nigeria and Pakistan. One study found that many obstetric patients at one of the trial sites–University College Hospital, Ibadan–received single unit blood transfusions despite the need for additional units, based on estimated blood loss or low hematocrit values [48,49]. Similarly, in Pakistan, a 40% shortage of blood and blood components limits the amount of donated blood available for transfusion [50]. The higher frequency of single unit transfusions in low income countries may be driven by both lower blood donation rates and the higher frequency of anemia in low income countries. While restrictive transfusion practices in a range of settings have been shown to be beneficial, single unit transfusions may be insufficient to treat patients suffering from severe traumatic and postpartum hemorrhage [51–53].

Both increased time from trauma to hospitalization and increased time from childbirth to hospitalization increased the likelihood of transfusion. One explanation for these results may be that indicators of extended hypoperfusion, like tissue ischemia, are unaccounted for by measuring traditional hemodynamic factors such as blood pressure, leading to residual confounding. We also found that Caesarean section was associated with an increased risk of blood transfusion. One possible reason for this observation may be that Caesarean sections tend to be performed during more complicated pregnancies, where hemodynamic stability is compromised by additional pathology. Another possibility is that Caesarean sections are necessary due to insufficiently controlled bleeding. Factors that increase the likelihood of Caesarean section include placenta previa, fetal distress, breech presentation, and macrosomia [54,55]. We also found evidence that identifiable causes of hemorrhage other than uterine atony, including surgical trauma or tears, placenta previa or accreta, and other causes of hemorrhage, increased the likelihood of blood component transfusion. Differences in transfusion likelihood amongst causes of hemorrhage may relate to the effectiveness of managing bleeding arising from different causes. For example, for postpartum hemorrhage due to uterine atony, uterotonics are a first line treatment, and sometimes transfusion may be avoided by successful uterotonic intervention [56,57].

## Conclusions

Our study found that, for patients who suffered from traumatic hemorrhage, risk factors for transfusion were time since injury, country income level, and hemodynamic factors, including systolic blood pressure and heart rate. Patients with postpartum hemorrhage have an increased likelihood of transfusion with childbirth outside of the hospital, increased time since giving birth, Caesarean section, birth in lower-middle income countries, and all identifiable causes of hemorrhage other than uterine atony. Hemodynamic factors, including systolic blood pressure, estimated blood loss, and clinical signs of hemodynamic instability, were also associated with increased likelihood of transfusion in postpartum hemorrhage patients. The findings of this study have clinical implications. Altering modifiable factors, by reducing time from injury or childbirth to hospitalization, could reduce blood transfusions and complications associated with transfusions. Future studies should investigate subgroup analyses of the various factors that contribute to the association between risk factors and transfusion.

## Supporting information

S1 Data (DOCX)

## Abbreviations

CI: Confidence interval
CRASH-2: Clinical Randomisation of an Antifibrinolytic in Significant Hemorrhage-2
WOMAN: World Maternal Antifibrinolytic Trial
ARR: Adjusted risk ratio
RR: Risk ratio
