# Supplementary material for: Risk factors for blood transfusion in traumatic and postpartum hemorrhage patients: Analysis of the CRASH-2 and WOMAN trials
Source: PLoS One. 2020 Jun 3;15(6):e0233274. doi: 10.1371/journal.pone.0233274 (PMC7269233; doi:10.1371/journal.pone.0233274)
Supplement: S1 Data — (DOCX) [file pone.0233274.s001.docx]

# Supporting information

For the CRASH-2 trial, countries were categorized by income level according to the World Bank 2017 classification system as follows:

- high-income – Australia, Belgium, Canada, Czech Republic, Italy, Japan, Saudi Arabia, Singapore, Slovakia, Spain, and United Kingdom
- upper-middle-income – Albania, Argentina, China, Colombia, Cuba, Ecuador, Iran, Iraq, Jamaica, Malaysia, Mexico, Peru, Serbia, South Africa, and Thailand
- lower-middle-income – Bangladesh, Cameroon, Egypt, El Salvador, Georgia, Ghana, India, Indonesia, Kenya, Nigeria, Sri Lanka, Tunisia, and Zambia
- low-income – Tanzania

For the WOMAN trial, country income level, was classified according to the World Bank 2017 classification system as follows:

- high-income – United Kingdom
- upper-middle-income – Albania, Colombia, and Jamaica
- lower-middle-income – Bangladesh, Cameroon, Egypt, Ghana, Kenya, Nigeria, Pakistan, Papua New Guinea, Sudan, and Zambia
- low-income – Burkina Faso, Democratic Republic of the Congo, Ethiopia, Nepal, Tanzania, and Uganda
